# Investigation of the Structural, Mechanical and Operational Properties of an Alloy AlSi18Cu3CrMn

**DOI:** 10.3390/ma18235434

**Published:** 2025-12-02

**Authors:** Desislava Dimova, Boyan Dochev, Karel Trojan, Kalina Kamarska, Yavor Sofronov, Mihail Zagorski, Veselin Tsonev, Antonio Nikolov

**Affiliations:** 1 Center of Competence “Smart Mechatronic, Eco-and Energy-Saving Systems and Technologies”, 4000 Plovdiv, Bulgaria; 2 Department of Mechanical Engineering and Technologies, Faculty of Mechanical Engineering, Technical University of Sofia, Branch Plovdiv, 4000 Plovdiv, Bulgaria; kamarska@tu-plovdiv.bg; 3 Department of Solid State Engineering, Faculty of Nuclear Sciences and Physical Engineering, Czech Technical University in Prague, 12000 Prague, Czech Republic; 4 Department of Theory of Mechanisms and Machines, Faculty of Industrial Technology, Technical University of Sofia, 1756 Sofia, Bulgaria; ysofronov@tu-sofia.bg (Y.S.); mihail.zagorski.tu@gmail.com (M.Z.); 5 Department of Mechanics, Faculty of Transport, Technical University of Sofia, 1756 Sofia, Bulgaria; tzonev@tu-sofia.bg; 6 Department of Material Science and Technology of Materials, Faculty of Industrial Technology, Technical University of Sofia, 1756 Sofia, Bulgaria; anikolov@tu-sofia.bg

**Keywords:** aluminum–silicon alloy, structure, mechanical properties, phase composition, performance properties

## Abstract

A non-standardized hypereutectic aluminum–silicon alloy, AlSi18Cu3CrMn, was developed. To refine the structure of the studied composition, a phosphorus modifier was used in an amount of 0.04 wt %, and a complex modifying treatment was applied by combining the chemical elements of phosphorus, titanium, boron and beryllium (P, 0.04 wt %; Ti, 0.2 wt %; B, 0.04 wt %; Be, 0.007 wt %). To improve the mechanical and operational properties of the alloy, it was heat-treated (T6) at a temperature of 510–515 °C before quenching, with artificial aging applied at a temperature of 210 °C for 16 h. Phosphorus-modified alloy AlSi18Cu3CrMn was quenched in water at 20 °C, and the combined modified alloy was quenched in water at temperatures of 20 °C and 50 °C. By conducting a microstructural analysis, the free Si crystals and silicon crystals in the composition of the eutectic in the alloy structure were characterized, and by conducting XRD, the presence and type of secondary phases were established. The hardness of the alloy was measured, as well as the microhardness of the α-solid solution. Static uniaxial tensile testing was carried out at normal and elevated temperatures (working temperatures of 200 °C, 250 °C and 300 °C). By using a gravimetric method, the corrosion rate of the alloy in 1 M NaCl and 1 M H_2_SO_4_ was calculated. The mass wear, wear intensity and wear resistance of the studied AlSi18Cu3CrMn alloy were determined during reversible reciprocating motion in the boundary-layer lubrication regime.

## 1. Introduction

The piston is one of the most critical and highly loaded components in internal combustion engines. Its primary functions include sealing the combustion chamber and participating in the conversion of thermal energy generated during the combustion process into mechanical work through its interaction with the crankshaft–connecting rod mechanism. The piston is subjected to various forces, including gas pressure forces, inertial forces, mass forces and frictional forces. It experiences intense thermal loading due to direct contact with high-temperature combustion gases, in addition to mechanical loading from friction. As a result of elevated temperatures and limited lubrication, the piston undergoes mechanical, abrasive and corrosive wear. Under combined mechanical and thermal loading, complex stress and deformation fields are generated within the piston, which significantly affect the reliability and service life of the engine. The combined effect of these loads can lead to piston degradation manifested as material fatigue, cracking, plastic deformation and wear in the contact zones with the cylinder liner. In recent years, researchers have focused on reducing emissions from internal combustion engines by using alternative fuels [1,2] and increasing their reliability by optimizing existing materials or developing new material solutions.

Currently, eutectic alloys of the Al-Si system are used to manufacture components for friction pairs in automobile engines. In recent years, leading manufacturers have imposed hypereutectic alloys of the Al-Si system as a material for piston manufacturing, despite their deteriorated technological properties (compared to eutectic alloys). The main reasons for this include the structure of this type of alloy, which provides less mass loss during the operation of the pistons, as well as less expansion of the alloys at elevated operating temperatures in engines [3,4,5,6].

The alloying elements used in the composition of piston silumins have a significant impact on their structure and phase composition, as well as on their mechanical and operational properties [7,8]. The most commonly used alloying elements are Mg, Cu and Ni [5,6]. Magnesium (Mg) interacts with silicon (Si), forming the Mg_2_Si phase, and participates in the composition of other complex intermetallic compounds after T6 heat treatment, positively affecting the strength of the alloys [9,10,11,12]. Alloying of aluminum alloys with copper (Cu) improves the mechanical properties of the alloys [13,14,15,16,17] because after T6, the CuAl_2_ phase is formed and separated. Alloying with nickel (Ni) is carried out to increase the mechanical strength and improve the operational properties of aluminum alloys, and it also has a positive effect on the plasticity of the alloys after heat treatment, the reason being the strengthening of the solid solution [18,19,20,21]. It is necessary to note that at a concentration of Ni above 1 wt % in the composition of alloys, a decrease in the strength of the alloys was recorded due to agglomeration of nickel-rich compounds [22].

Iron (Fe) in an amount above 0.4 wt % is a harmful impurity in the composition of aluminum alloys due to the formation of chemical compounds with high microhardness, irregular shape and large size, which act as stress concentrators and deteriorate the properties of the alloys [23]. To eliminate the negative effect of iron-containing phases, alloys are additionally alloyed with manganese (Mn), chromium (Cr), cobalt (Co), beryllium (Be), molybdenum (Mo), nickel (Ni), vanadium (V), tungsten (W), strontium (Sr), as well as the rare earth elements cerium (Ce), lanthanum (La), neodymium (Ne) and yttrium (Y) [23,24]. Due to their limited solubility in aluminum, refractory elements Cr, Mo, V and W are less often used for the alloying of Al-Si alloys, but they can be useful in strengthening alloys after heat treatment. The use of these elements in the composition of alloys intended for the production of parts in sand or ceramic molds is undesirable because these elements form intermetallics, which make the alloy brittle at lower cooling rates in the crystallization process. It is recommended to use them when casting in metal molds, i.e., in a technological process with guaranteed high cooling rates, which, in turn, causes the formation of a supersaturated α-phase and improves the properties of the alloys [25]. Of particular importance for eliminating the undesirable impact of iron (Fe) on the quality of piston silumins is the accurate dosing of the concentration of the above-mentioned chemical elements. Typically, the most commonly used element is manganese (Mn), and it is recommended that the quantitative ratio of Mn:Fe be at least 0.5, especially if the melt is contaminated with iron and its concentration in the alloy composition is greater than 0.45 wt % [26]. The introduction of larger amounts of Mn into the alloy composition is a prerequisite for the deterioration of its mechanical properties due to the formation of larger amounts of chemical compounds, the composition of which includes both iron (Fe) and manganese (Mn) [27]. There is evidence that higher chromium content (similar to manganese) is the basis for a decrease in the quality of alloys due to the formation of significantly larger amounts of iron-containing intermetallics in the composition [28,29,30]. When the amount of chromium used is such that its ratio relative to iron (Cr:Fe) is greater than 0.33, the effect of its introduction into the alloy composition is greatest [24]. It has been established that the positive influence of chemical element chromium (Cr) on the properties of alloys occurs after heat treatment [21,31,32,33,34,35].

The resulting structure influences the production of aluminum–silicon alloys with increased mechanical strength and improved performance properties. Of particular importance is the metallurgical treatment of the melts (refining, degassing and modification). Eutectic aluminum–silicon alloys are usually modified with sodium (Na) [36,37,38], but in recent years, the possibilities of using strontium (Sr) [38,39,40,41,42] and antimony (Sb) [21,36,43,44] have been considered. These modifiers have a positive effect on the mechanical mixture (eutectic) by giving it a finely dispersed form [36]. Elements titanium (Ti) and boron (B) are used to modify the α-phase in the structures of hypoeutectic silumins, and their modifying effect is due to the formation of Al_3_Ti and TiB_2_ compounds [45]. To introduce modifiers Ti and B into the melts of these alloys, ligatures based on Al-Ti-B are most often used [37]. There is evidence that the use of ligature Al-Ti5-B1 also has a positive effect on the free silicon crystals in the structures of the hypereuteric silumins [46,47]. The structure of hypereuteric aluminum–silicon alloys is a kind of natural composite in which free silicon crystals with high microhardness are located in a soft and plastic matrix. Typically, unmodified Si crystals are irregularly shaped and large in size (80–150 µm); they act as stress concentrators, which is the reason for the deterioration of the properties of the alloys [7,48,49,50,51]. The same negative impact is exerted by eutectic silicon crystals when they are unmodified and are large in size and in the form of needle-like plates [52,53]. This requires the application of a modifying treatment aimed at reducing the free silicon crystals and giving them a polygonal shape, as well as the refinement and rounding of silicon crystals in the eutectic matrix, which, in turn, leads to an increase in the mechanical properties of the alloys [54,55,56,57,58,59]. The most commonly used modifiers are phosphorus and sulfur [60,61,62,63,64,65] because they interact with aluminum and form the AlP and AlS compounds, characterized by a high melting point, with a crystal lattice of the same type and with parameters close to those of silicon crystals, i.e., crystallization centers on which silicon crystals can form and grow [66,67,68,69]. Chemical element phosphorus (P) is mainly used to modify free silicon crystals due to the formation of AlP [70,71,72], but its use has been found to affect the surface tension at the grain boundary [73,74,75,76,77]. Based on the fact that some modifiers work as surfactants and inoculants (first- and second-order modifiers) [78,79], it can be assumed that phosphorus is adsorbed at the boundaries of growing α-crystals in the composition of the eutectic, blocking their growth, i.e., it also works as a first-order modifier (surfactant).

The aims of the present work are to compose a new (non-standardized) hypereutectic aluminum–silicon alloy, AlSi18Cu3CrMn; to carry out modification with phosphorus (P) and combined modification with P, Be, Ti and B; and to investigate the structural, phase composition, mechanical and performance properties of the alloy.

## 2. Materials and Methods

The object of the current research is hyperdeutectic aluminum–silicon alloy AlSi18Cu3CrMn, the chemical composition of which is indicated in Table 1.

The preparation of the studied alloy is a two-stage process including the preparation of the alloy from technically pure Al, Si and Cu metals, with only the intended amount of Mn being introduced into the alloy composition by using the Cu70Mn30 alloy, casting of the block, and subsequent melting and metallurgical treatment of the melts. The reason for using this approach is the results of previous studies [82], which show that when the phosphorus modifier is introduced at high temperatures (930–950 °C), there is a possibility that the formed AlP inoculants can grow to such sizes that the formation and growth of silicon crystals is difficult and the resulting structures are not sufficiently well modified, i.e., the positive modifying effect of phosphorus is reduced.

The first stage of alloy preparation includes melting the aluminum, overheating it to 950 °C and adding the calculated amount of silicon; after its complete dissolution, in the aluminum, the calculated amounts of copper and the Cu70Mn30 alloy are added (again, at a melt temperature of 950 °C). The melting process is carried out in an electric resistance furnace produced from “Enginex” LTD Plovdiv, Bulgaria under a layer of coating-refining flux produced from “Telemat-Trade” LTD Sofia, Bulgaria (10 KCl : 50 NaCl : 10 Na_3_AlF_6_) in an amount of 0.5 wt % of the alloy mass. This is followed by the casting of the alloy in the form of a block.

The second stage of alloy preparation involves remelting the pre-cast block in a laboratory electric resistance furnace with a graphite crucible again under a layer of covering-refining flux (10 KCl : 50 NaCl : 10 Na_3_AlF_6_) in an amount of 0.5 wt % of the alloy mass. After reaching a temperature of 760 °C, the calculated amount of flux-ligation Al20Cr80 is added to the melt to introduce the alloy and the chromium alloying element into the composition. When modifying the alloy with phosphorus in an amount of 0.04 wt %, the same is introduced into the melt through the phosphorus copper ligature (CuP10) in an amount of 0.4 wt % of the alloy mass at a temperature of 830 °C. After complete absorption of the modifier, the slag is cleaned, and the melt is degassed with argon for 3–5 min. After degassing, the surface of the melt is cleaned again, and experimental castings are cast at a temperature of 800 °C.

To study the influence of the complex modifying treatment on the structure of the AlSi18Cu3CrMn alloy with modifiers P, Ti, B and Be modifiers in amounts of 0.04 wt %, 0.2 wt %, 0.04 wt % and 0.007 wt %, respectively, the melting process was carried out under the same conditions (as in the modification with phosphorus), and the modifying treatment was, again, applied at a temperature of 830 °C. The concentrations of the alloying elements were selected as optimal based on preliminary experimental studies conducted with varying beryllium modifier contents [83], as well as on prior results obtained for phosphorus [84]. First, the CuP10 ligature was introduced in an amount of 0.4 wt % of the alloy mass, followed by the introduction of the calculated amount of Be using beryllium bronze (CuCo1Ni1Be). The necessary amounts of the Ti and B modifiers were introduced through the AlTi5B1 ligature. After their absorption, the slag was cleaned, and the melt was degassed with argon for 3–5 min. After degassing, the surface of the melt was cleaned again, and experimental castings were cast at a temperature of 800 °C. Immediately before casting the experimental castings, the melt was intensively stirred with a graphite stirrer to prevent the precipitation of the formed borides.

For experimental castings intended for the production of test specimens for the planned research, metal casting equipment was used, pre-coated and heated to a temperature of 200 °C.

To determine the chemical composition of the studied alloys, spectral analysis of the studied alloys was carried out using an Oxford Instruments FOUNDRY-MASTER UV apparatus [85].

The experimental castings were subjected to T6 heat treatment, in which the structure was heated at a temperature of 510–515 °C for homogenization; the holding time at this temperature was 6.5 h, and water at temperatures of 20 °C and 50 °C was used as a cooling medium, ensuring different cooling rates. After quenching of the test bodies, artificial aging was carried out at a temperature of 210 °C, and the holding time at the selected operating temperature was 16 h. The heat treatment was carried out with a chamber laboratory electric resistance furnace (model F20 TE11 of “Techeco”, Sofia, Bulgaria).

To study the influence of the modification treatment on the structure of the studied alloy after heat treatment, a microstructural analysis was carried out. The preparation of the microsections for metallographic analysis was carried out according to a standard methodology: wet grinding on abrasive paper with an increasing number from № 240 to № 1000 and polishing with diamond paste and lubricant until a mirror surface of the sections was obtained. The microstructure of the sections prepared as such was revealed with Keller’s reagent (1 part HF, 1.5 parts HCl, 2.5 parts HNO_3_ and 95 parts H_2_O) and brightened with HNO_3_. The study was carried out on a Leica DM ILM microscope with the help of software.

To study the influence of the used alloying elements and modifiers, a phase analysis of the alloy was also conducted. An X’Pert PRO MPD diffractometer (Malvern Panalytical B.V., Almelo, The Netherlands) with cobalt radiation was used to obtain X-ray diffraction (XRD) patterns [85].

To conduct the static uniaxial tensile test at room temperature, standardized test specimens were manufactured, and the test was performed on a “Zwick/Roell Z 250” universal tensile testing machine according to the BDS EN ISO 6892-1:2020 standard [86]. Values for the tensile strength (R_m_), the conditional yield strength (R_p_) and the relative elongation (A_5_) of the alloys were recorded. The values of the mechanical characteristics were averaged based on the test results of 6 ÷ 8 test specimens. Tensile tests were conducted at elevated temperatures in accordance with the BDS EN ISO 6892-2:2018 standard [87]. The test consisted of heating the test specimen to the specified temperature and loading it with a tensile force until failure in order to determine the tensile strength (R_m_). The obtained results are average values from the test of 6 ÷ 8 test specimens.

The tribological characteristics of the studied alloys were determined by wear tests in the boundary lubrication regime, carried out with a tribotester RT16 in reciprocating motion. Contact between the sample and the counter-body was realized under strictly controlled kinematic and loading parameters, ensuring constant friction conditions. The main wear indicators—mass loss, intensity and absolute wear resistance—were measured through successive cycles of weighing, cleaning and thermal drying of the samples with high accuracy. The counter-body was made of S235JR steel with a hardness of 130HV, and lubrication was provided by a drip system with a constant flow rate. All tests were carried out under identical conditions of load, sliding speed and friction path, which allowed for a direct comparison of the wear resistance of the differently modified alloys.

Corrosion tests of the alloy were also carried out, which consisted of determining the weight loss of the tested alloys exposed to 1 M NaCl and H_2_SO_4_ solutions. Before testing, the samples were immersed in ethyl alcohol for 5 min, washed with distilled water, dried and weighed on an Acculab ATILON analytical balance with an accuracy of ±0.0001 g. Then, the samples were placed in 1 M NaCl solution and 1 M H_2_SO_4_ solution at room temperature. The first measurement was conducted after 72 h, and the following measurement was taken after 144 h, with the maximum testing period being 360 h. After each testing period, the AlSi18Cu3CrMn samples were cleaned with a brush under running water, dried and weighed. For each of the testing periods of the samples, in the corresponding corrosion environment, the mass loss and corrosion rate were calculated. From the obtained CR values, conclusions are drawn about the corrosion behavior of the alloy.

The macrohardness of the studied alloy was also measured using the Brinell method, using a steel indenter (sphere) with a diameter of 2.5 mm, a pressing force of 62.5 kg and a holding time under load of 30 s. The measurement was performed using a Brinell FOUNDRAX BRIN400D hardness tester. The microhardness of the α-phase was also measured using a Vickers HV-1000 hardness tester. The measurement was performed with a load of 50 g and a holding time of 10 s.

## 3. Results

**Figure 1:**
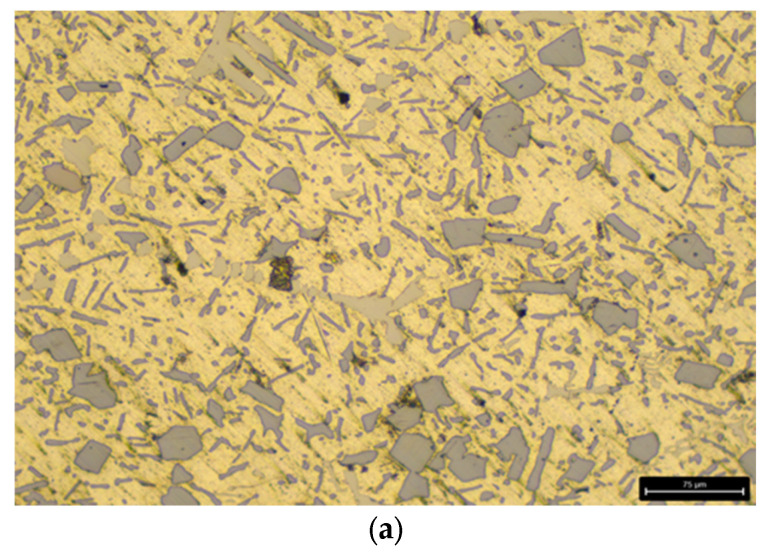

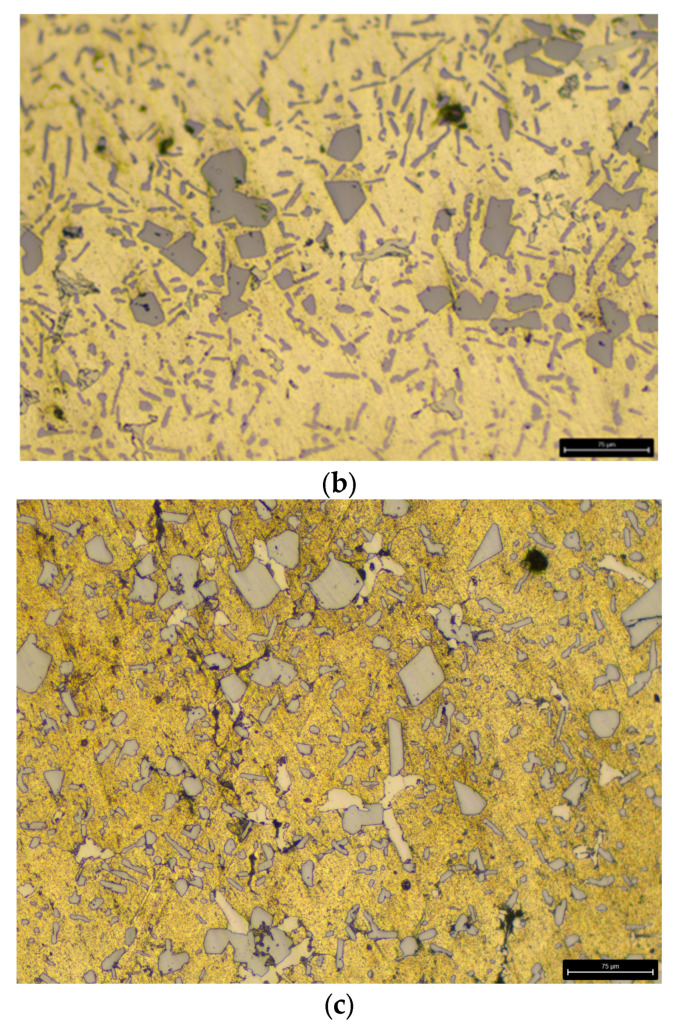
Microstructure of the studied alloys at low magnification. (**a**) AlSi18Cu3CrMn alloy with 0.04 wt.% P, subjected to T6 heat treatment. (**b**) AlSi18Cu3CrMn alloy with P, Ti, B and Be subjected to T6 heat treatment (quenched in water at 20 °C). (**c**) AlSi18Cu3CrMn alloy with P, Ti, B and Be subjected to T6 heat treatment (quenched in water at 50 °C) [83].

**Figure 2:**
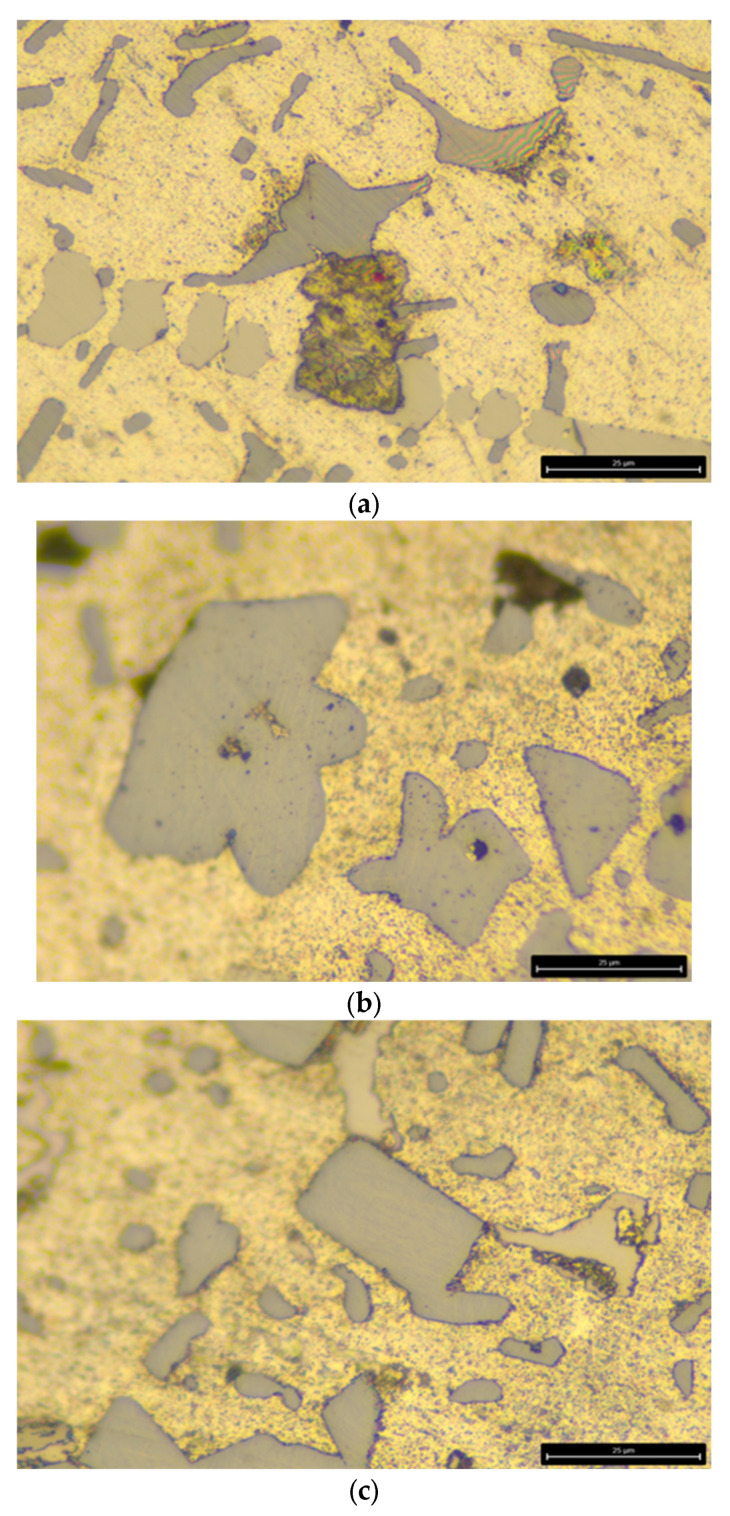
Microstructure of the studied alloys at high magnification. (**a**) AlSi18Cu3CrMn alloy with 0.04 wt.% P, subjected to T6 heat treatment. (**b**) AlSi18Cu3CrMn alloy with P, Ti, B and Be subjected to T6 heat treatment (quenched in water at 20 °C). (**c**) AlSi18Cu3CrMn alloy with P, Ti, B and Be subjected to T6 heat treatment (quenched in water at 50 °C) [83].

After applying the heat-treatment regime of the AlSi18Cu3CrMn alloy, a metallographic analysis was carried out on both the alloy modified with phosphorus only and the complex-modified alloy (P, Ti, B and Be), in which two cooling rates were used during quenching, with water at temperatures of 20 and 50 °C acting as the cooling medium. The microstructural analysis determined the characteristics of the shape and size of free silicon and silicon in the composition of the eutectic alloy. The obtained data were statistically processed and presented as average values. Figure 1a and Figure 2a show the microstructure of the AlSi18Cu3CrMn alloy modified with phosphorus with a concentration of 0.04 wt %, subjected to T6 heat treatment, with the quenching carried out in water at a temperature of 20 °C.

The microstructure of the alloy modified only with phosphorus consists of a eutectic mixture and primary separated silicon crystals. The free Si crystals in the observed field of the metallographic section are of rounded and polygonal in shape, with straight walls and a conditional average diameter in the range of 35–40 µm. The silicon crystals in the composition of the eutectic alloy are summarized in two groups. The first group is of rounded shape, with a conditional average diameter of 8 µm, and the second group is of “needle” shape, with an average linear dimensions of 40 µm.

The microstructure of the studied alloy modified with 0.04 wt % P, 0.2 wt % Ti, 0.04 wt % B and 0.007 wt % Be subjected to heat treatment according to the T6 regime, quenched in water at a temperature of 20 °C, is shown in Figure 1b and Figure 2b. The primary silicon crystals are highly refined, with a conditional average diameter of 31 µm. The silicon crystals included in the eutectic structure have a plate-like shape and, in the field of the micrograph, are observed as “needles” with linear dimensions, again summarized in two size groups: from 3 to 7 µm and from 10 to 30 µm.

The free silicon crystals in the structure of the AlSi18Cu3CrMn alloy modified with P, Ti, B and Be, subjected to the T6 regime, water quenching at a temperature of 50 °C and artificial aging, as in the previous two studies (heating temperature of 210 °C and holding time of 16 h) are of a rounded shape, with a conditional average diameter in the range of 36–40.2 µm (Figure 1c). In the composition of the eutectic alloy, small silicon crystals are also observed, with sizes in the range of 7–12 µm and of a rounded shape (Figure 2c). In the observed field of the metallographic section, large intermetallic phases are observed, the type of which will be clarified by X-ray structural analysis.

In the phase analysis of the AlSi18Cu3CrMn alloy, it was found that the main intermetallics (CuAl_2_, Cr_3_Si, Mn_12_Si_4_ and Al_57_Mn_12_) are stable and are registered in all three studied samples. The (Si,Al)_2_Cr phase was registered only in the alloy modified with P, Ti, B and Be, subjected to heat treatment in the T6 regime with a coolant temperature of 50 °C during quenching. The higher cooling temperature provides a lower cooling rate, which leads to an increase in the diffusion and separation of Cr-containing phases. Table 2 shows the results of the phase analysis of the alloy sample № 1 AlSi18Cu3CrMn modified only with phosphorus, subjected to the T6 regime with a 20 °C cooling medium during quenching. Sample № 2 is the alloy modified with P, Ti, B and Be, subjected to the T6 regime with a quenching water temperature of 20 °C, and sample № 3 is the complex-modified alloy, again subjected to T6 but with a quenching coolant temperature of 50 °C.

**Figure 3:**
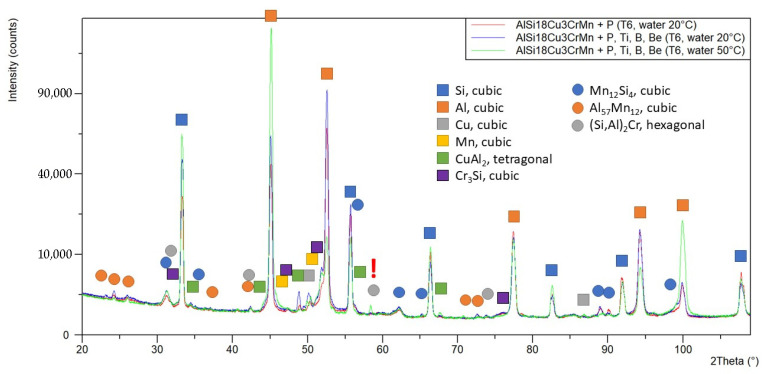
Comparison of diffraction diagrams of samples.

Figure 3 shows the comparative results of the phase analysis of the three alloys.

The results of the mechanical tests performed at room and elevated temperatures, as well as the measured macro- and micro-hardness, are summarized in Table 3.

The mechanical properties of the AlSi18Cu3CrMn alloys are determined by the complex interaction between the alloying elements and the conditions of the T6 heat treatment (different cooling rates). The optimal combination of high strength and hardness was achieved in the alloy containing only phosphorus quenched at 20 °C, where we recorded the highest tensile strength and the highest microhardness of the α-phase. With the addition of the P, Ti, B and Be modifiers, a decrease in the tensile strength (R_m_) is observed, and in sample № 3, with a decrease in the cooling rate during quenching, the lowest microhardness of the studied alloys was also recorded.

Given the fact that the studied alloys are mainly used in the manufacture of pistons for internal combustion engines operating under increased thermal loads, testing of the mechanical properties at high temperatures was also carried out. Considering that both the microstructural analysis and the results of the phase study show more favorable structural characteristics and phase composition for samples quenched in water at a temperature of 20 °C, tensile tests at elevated temperatures were carried out only on alloys cooled with a higher cooling rate during quenching. Three specimens of each studied alloy were tested at temperatures of 200 °C, 250 °C and 300 °C. The specified loading rate was 6 MPa/s. Table 3 show the obtained experimental results.

The results of the high-temperature tests show a clear increase in the tensile strength of the alloy modified with phosphorus, titanium, boron and beryllium, while in room-temperature tests, this alloy demonstrates lower values of tensile strength (Rₘ) compared to the alloy modified with phosphorus only. When the temperature is increased to 200 °C, a significant increase in strength is observed, and even at 300 °C, the values remain relatively high. This behavior indicates good structural and phase stability properties of the material under thermal stress. It is assumed that the presence of Ti, B and Be leads to the formation of dispersed and thermally stable intermetallic particles, which inhibit grain growth and limit the coalescence of the silicon phase at elevated temperatures.

The results of the tribological study of friction in the boundary lubrication regime (15W40 oil) show distinct differences in the behavior of the three studied AlSi18Cu3CrMn alloys depending on the composition and the quenching temperature (Table 4). For the alloy modified only with phosphorus and quenched in water at a temperature of 20 °C, mass-wear values of the sample of 0.4 mg and of the counterbody of 5.1 mg were measured. The total mass loss for the tribosystem reaches 5.5 mg, which corresponds to a wear intensity of 4.9 × 10^−4^ mg/(m·N) and a wear resistance of 2.0 × 10^3^ (m·N)/mg. These values indicate relatively good wear resistance but with higher wear of the steel counterbody, which suggests a rougher contact and an unstable boundary layer.

The addition of the Ti, B and Be modifiers to the alloy (at the same quenching temperature of 20 °C) leads to a clear improvement in the tribological behavior. The mass loss of the sample decreases to 0.3 mg, and that of the counterbody decreases to 3.0 mg, which corresponds to a total wear of 3.3 mg—about 40% lower compared to the unmodified alloy. The wear intensity decreases by almost twofold (2.5 × 10^−4^ mg/(m·N)), while the wear resistance doubles to 4.0 × 10^3^ (m·N)/mg. This improvement can be explained by the influence of the Ti, B and Be modifiers on microstructural refinement and the increased adhesion resistance of the surface layer.

Increasing the quenching temperature to 50 °C for the complex-modified alloy leads to a slight increase in the wear of the sample (0.4 mg) but a significant decrease in the wear of the counterbody (0.9 mg). The total wear of the tribosystem drops to 1.3 mg—the lowest value among all variants. The wear intensity decreases to 3.3 × 10^−4^ mg/(m·N) for the alloy and 7.4 × 10^−4^ mg/(m·N) for the counterbody, and the corresponding wear resistance values increase to 3.0 × 10^3^ and 1.4 × 10^3^ (m·N)/mg. This indicates that the moderate decrease in the quenching cooling rate (50 °C water) led to the formation of a more resistant surface structure, probably due to the partial separation of CuAl_2_ and (Si,Al)_2_Cr phases, which act as dispersed strengthening components. The results of the conducted tribological studies are summarized in Table 4 and Table 5.

The corrosion behavior of AlSi18Cu3CrMn alloys modified with various modifiers was investigated by the gravimetric method in two corrosion media—1 M NaCl and 1 M H_2_SO_4_. The results are presented as the corrosion rate (CR, g/(m^2^·h)) considering the results for 72, 240 and 360 h shown in Table 6 and Table 7.

In a neutral environment (1 M NaCl) with water quenching at 20 °C, the alloy containing only phosphorus shows extremely high corrosion resistance—no mass loss is observed up to 240 h, and only after 360 h is a minimum corrosion rate of 0.00015 g/(m^2^·h) recorded. The complexly modified alloy with P, Ti, B and Be at the same quenching temperature exhibits slightly higher initial corrosion activity (0.0014 g/(m^2^·h) for 72 h), but the values level out with continued holding. When quenching in an aqueous environment at 50 °C, the lowest total corrosion rate is observed, with the value at 360 h being only 0.00014 g/(m^2^·h). These results show that all alloys exhibit excellent resistance in chloride-containing environments, with the low mass loss values for the sample quenched in water at 50 °C likely due to a more compact and stable passive film.

In acidic medium (1 M H_2_SO_4_), a more intense mass loss is observed, which is expected for aluminum alloys. Corrosion rates are approximately twice as high as those in NaCl. However, the values for the three alloys remain close—in the range of 0.012–0.013 g/(m^2^·h), with slightly lower values observed for the alloy modified with P, Ti, B and Be and quenched at 20 °C (0.0123 g/(m^2^·h) after 240 h). This indicates that complex modification contributes to improved resistance to the aggressive effects of the acidic medium, probably due to the refinement of the microstructure and stabilization of the intermetallic boundaries.

**Figure 4:**
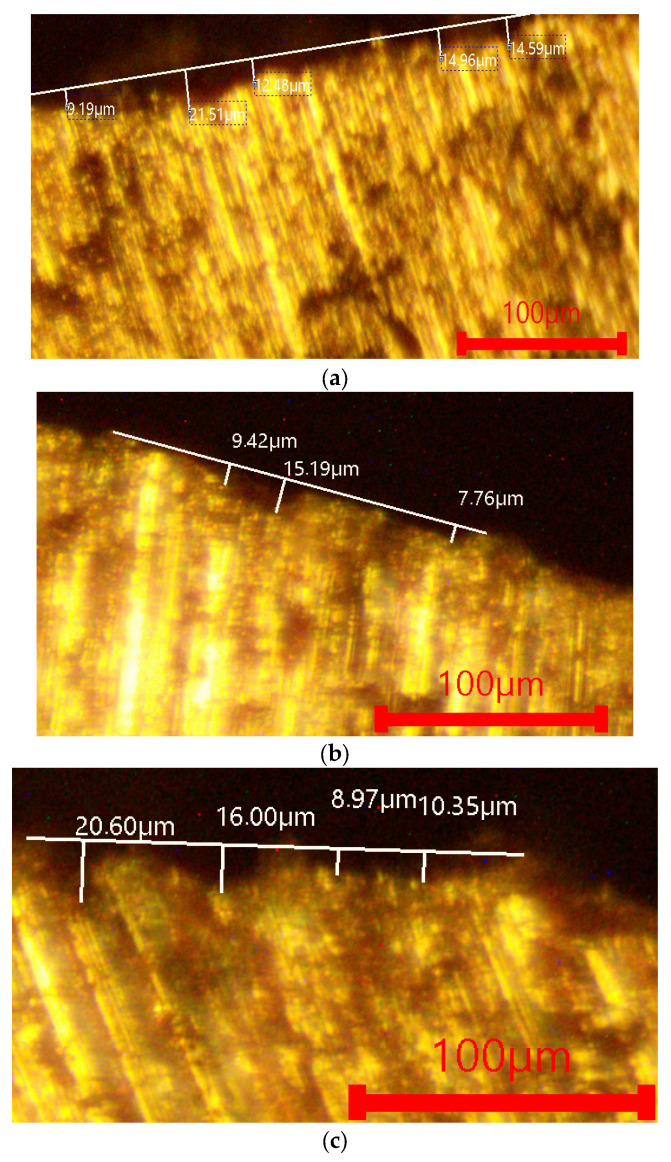
Optical metallography of the investigated alloys after immersion in 1 M NaCl. (**a**) AlSi18Cu3CrMn alloy with 0.04 wt.% P, subjected to T6 heat treatment. (**b**) AlSi18Cu3CrMn alloy with P, Ti, B and Be, subjected to T6 heat treatment (quenched in water at 20 °C). (**c**) AlSi18Cu3CrMn alloy with P, Ti, B and Be, subjected to T6 heat treatment (quenched in water at 50 °C).

**Figure 5:**
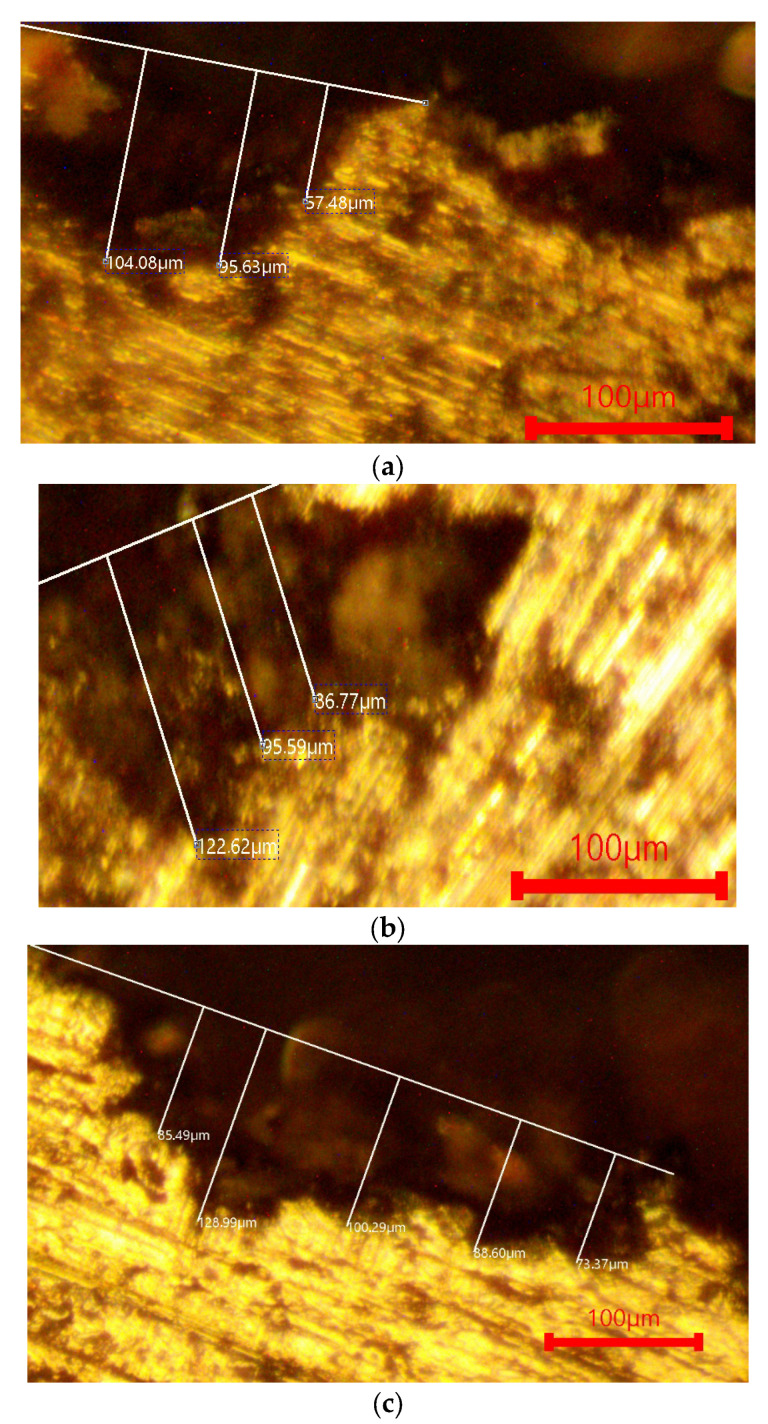
Optical metallography of the investigated alloys after immersion in 1 M H_2_SO_4_. (**a**) AlSi18Cu3CrMn alloy with 0.04 wt.% P, subjected to T6 heat treatment. (**b**) AlSi18Cu3CrMn alloy with P, Ti, B and Be, subjected to T6 heat treatment (quenched in water at 20 °C). (**c**) AlSi18Cu3CrMn alloy with P, Ti, B and Be, subjected to T6 heat treatment (quenched in water at 50 °C).

Figure 4 and Figure 5 present characterized microstructural images of the surface of the investigated alloys after immersion in 1 M NaCl and 1 M H_2_SO_4_. The images visualize the surface relief and the degree of material loss. In the NaCl solution (Figure 4), all alloys exhibit minimal corrosion attack, which corresponds to the very low corrosion rates. The penetration depth is shallow, and the surface retains its integrity, indicating the formation of a stable passive oxide film. The complex-modified alloy (P, Ti, B and Be) shows a smoother and more uniform surface.

In the 1 M H_2_SO_4_ environment (Figure 5), more pronounced signs of corrosion are observed, including localized dissolution and increased roughness. Nevertheless, corrosion penetration remains relatively limited, and the observed morphology is consistent with the small differences in corrosion rates.

## 4. Discussion

The results obtained from the microstructural, mechanical, tribological and corrosion studies allow for a comprehensive assessment of the influence of the phosphorus (P) modifier and the combination of P, Ti, B and Be on the properties of the AlSi18Cu3CrMn alloy.

Microstructural analysis showed that modification with the classical modifier for these alloys, i.e., phosphorus, leads to a noticeable refinement of the primary silicon crystals and partial rounding of the eutectic silicon. The addition of titanium, boron and beryllium at the indicated concentrations further enhances this effect, leading to the formation of finer and more evenly distributed free silicon crystals. These results are consistent with the literature data, according to which Al_3_Ti and TiB_2_ particles act as crystallization centers and beryllium limits the growth of coarse intermetallics. The resulting refinement of the structure has a positive effect on the mechanical and tribological characteristics, as it reduces the concentration of stresses and provides a more uniform distribution of loads in the matrix.

X-ray diffraction (XRD) analysis confirms the presence of intermetallic CuAl_2_, Cr_3_Si, Mn_12_Si_4_ and Al_57_Mn_12_ phases in all samples. Only in the alloy quenched in water at 50 °C was the appearance of the (Si,Al)_2_Cr phase observed, indicating that a lower cooling rate favors the diffusion of chromium and the formation of stable chromium-containing compounds. These phases increase the thermal stability and hardness of the α-phase but may slightly reduce its ductility.

The presence of thermally stable phases such as Cr_3_Si and (Si,Al)_2_Cr, as confirmed by XRD analysis, is likely to have a positive effect on the high-temperature properties of the alloy. It is assumed that the Cr_3_Si phase, owing to its high hardness and stable crystal lattice, may hinder the diffusion of copper and manganese and contribute to the maintenance of a fine microstructure under short-term thermal exposure. In turn, the (Si,Al)_2_Cr phase likely stabilizes the interfaces between the α-phase and silicon crystals, reducing local stresses and improving the material’s resistance during brief heating periods. Although the presented high-temperature tests were of limited duration and did not allow for definitive conclusions regarding the long-term behavior of these phases, the obtained results suggest that their presence contributes to improved thermal stability and mechanical performance of the alloy in the short term.

The results of mechanical tests at room temperature show that the alloy modified only with phosphorus has the highest tensile strength and microhardness. The fact that the X-ray structural analysis recorded the same phases for both alloys indicates that the differences in mechanical properties are not due to the phase composition but to their morphology and distribution. The shape, size and spatial arrangement of the intermetallic particles are probably the main reason for the observed differences in the tensile strength and behavior of the alloys during tests at elevated temperatures. This combination of stable intermetallic compounds and optimal microstructure provides stabilization of the α-phase and contributes to the combination of high hardness and good thermal resistance of the AlSi18Cu3CrMn alloys. CuAl_2_- and Mn-containing phases are typical strengthening intermetallics in Al–Si–Cu alloys; they increase the hardness and thermal stability by delaying the relaxation of internal stresses in the α-phase. Cr_3_Si and (Si,Al)_2_Cr are stable at high temperatures and limit the diffusion of copper and manganese, which prevents grain growth and stabilizes phase boundaries under prolonged thermal stress. The registration of the (Si,Al)_2_Cr phase in the sample, complexly modified with P, Ti, B and Be and quenched in water at a temperature of 50 °C, is an indicator of increased thermal stability and is a prerequisite for better mechanical characteristics at elevated temperatures, although high-temperature mechanical tests have not been conducted for this alloy. Given the obtained results, future high-temperature tests are planned. In the microstructural analysis, large, light-gray phases are observed, probably of the (Si,Al)_2_Cr type, whose uneven distribution and large sizes lead to unfavorable morphology of the structure and can neutralize the positive effect of the presence of the (Si,Al)_2_Cr phase.

Tribological studies in the boundary lubrication regime showed that the complex-modified alloy, when quenched in water at 50 °C, has the lowest wear intensity and the highest wear resistance. The total wear of the tribosystem is reduced to 75% compared to the alloy containing only phosphorus. This is due to its more homogeneous structure, the uniform distribution of silicon particles and the formation of a stable surface tribofilm during friction. Modification with Ti and Be probably contributes to the formation of a more compact oxide layer, which improves the mechanical resistance of the surface and resistance to adhesive wear.

Corrosion tests conducted by the gravimetric method show very good resistance of all alloys both in neutral (1 M NaCl) and acidic (1 M H_2_SO_4_) media. In NaCl solution and with prolonged retention (up to 360 h), corrosion rates are very low, as is typical of passivated aluminum alloys. The best behavior was recorded in the alloy modified with P, Ti, B and Be and quenched at 50 °C, in which a dense and stable passive oxide layer enriched with Al_2_O_3_ and Cr_2_O_3_ is formed. In acidic medium, corrosion rates are higher, but the differences between the alloys are insignificant. A similar trend was observed in previous studies [90,91], which examined the behavior of aluminum alloys from the Al–Si–Cu system modified with beryllium and transition metals in various aggressive environments. In [90], it was established that for AlSi18Cu5Mg alloy modified with Be and tested in 0.1 M H_2_SO_4_, the addition of beryllium leads to a significant decrease in the corrosion rate as a result of silicon phase refinement and a more uniform distribution of intermetallic compounds. This structural improvement limited the formation of micro-galvanic couples and facilitated the development of a protective Al_2_O_3_ film on the alloy surface. In a subsequent study [91], the beneficial effect of alloying elements such as Ni, Co and Mo on the corrosion behavior of hypereutectic Al–Si alloys tested in 1 M H_2_SO_4_ and 1 M HCl was confirmed. It was found that these elements stabilize the surface oxide layer and reduce the corrosion rate during prolonged exposure (up to 504 h), with the highest resistance observed for combined alloying with Ni + Co + Mo. The results obtained in the present work are consistent with these observations, confirming that the addition of Be and complex modifiers (P, Ti, B and Be) contributes to more effective passivation and lower corrosion activity in aggressive environments. This effect is likely due to the combined action of stable oxides (Al_2_O_3_ and Cr_2_O_3_) and finely distributed intermetallic phases, which limit localized anodic zones and suppress pitting processes.

## 5. Conclusions

The conducted investigations demonstrate that both the modifying treatment and the parameters of the heat-treatment regime have a significant influence on the structure and properties of the AlSi18Cu3CrMn alloy. Modification with phosphorus alone results in higher strength and hardness at room temperature, whereas complex modification with P, Ti, B and Be shows a tendency toward higher tensile strength values at elevated temperatures.

X-ray diffraction and microstructural analysis indicate the presence of stable intermetallic compounds (CuAl_2_, Cr_3_Si, Mn_12_Si_4_, Al_57_Mn_12_ and (Si,Al)_2_Cr), which contribute to the strengthening and stabilization of the α-phase. However, the uneven distribution and large sizes of some of these phases can lead to unfavorable morphology and limit their positive effect.

The complex-modified alloy demonstrates better wear resistance and corrosion resistance compared to the alloy modified only with phosphorus. These results are probably due to the formation of a stable and compact oxide layer enriched with Ti and Be, which acts as a barrier against the diffusion of oxygen and aggressive ions. This oxide film increases the contact resistance of the surface, limits adhesive wear and slows down the development of corrosion processes. The combination of a fine microstructure and the presence of stable intermetallic compounds ensures high operational reliability of complex-modified AlSi18Cu3CrMn alloys.

## Figures and Tables

**Table 1:** Chemical composition of alloy AlSi18Cu3CrMn, wt % [80,81].

Si	Fe	Cu	Mn	Mg	Cr	Ni	Zn	Ti	Al
18.50	0.25	3.12	0.76	0.01	1.0	0.01	0.13	0.002	rest

**Table 2:** Phase analysis results.

Phase	Alloy		
	№ 1	№ 2	№ 3
Si, cubic	✔	✔	✔
Al, cubic	✔	✔	✔
Cu, cubic	✔	✔	✔
Mn, cubic	✔	✔	✔
CuAl_2_, tetragonal	✔	✔	✔
Cr_3_Si, cubic	✔	✔	✔
Mn_12_Si_4_, cubic	✔	✔	✔
Al_57_Mn_12_, cubic	✔	✔	✔
(Si,Al)_2_Cr, hexagonal			✔

**Table 3:** Tensile test [88,89].

№	Alloy	Rm/MPa	Rm/MPa 200 °C	Rm/Mpa 250 °C	Rm/Mpa 300 °C	HB_2.5/62.5/30_	µHV_50/10_
1	AlSi18Cu3CrMn + P (T6, water 20 °C)	230	181	159	120	116	140.7
2	AlSi18Cu3CrMn + P, Ti, B, Be (T6, water 20 °C)	165	205	179	136	133	139
3	AlSi18Cu3CrMn + P, Ti, B, Be (T6, water 50 °C)	175	-	-	-	133	104.5

**Table 4:** Massive wear of the elements and the tribosystem when lubricating the boundary layer with 15W40 oil.

Denotement	Mass Before Wear, g	Mass After Wear, g	Mass Loss, mg		
AlSi18Cu3CrMn + P (T6, water 20 °C)	1.6515	1.6511	0.4	5.5	
Counterbody	26.7808	26.7757	5.1		
AlSi18Cu3CrMn + P, Ti, B, Be (T6, water 20 °C)	1.6376	1.6373	0.3	3.3	
Counterbody	30.9147	30.9117	3.0		
AlSi18Cu3CrMn + P, Ti, B, Be (T6, water 50 °C)	1.6991	1.6987	0.4	1.3	
Counterbody	30.9117	30.9108	0.9		

**Table 5:** Characteristics of wear and wear resistance of elements and the tribosystem when lubricating the boundary layer with 15W40 oil.

Denotement	Wear, mg	Wear Intensity, mg/(m·N)	Wear Resistance, (m·N)/mg	Wear-induced Mass Loss (mg)			
				Mass loss, mg	Wear Rate, mg/(m·N)	Wear Resistance, (m·N)/mg	
AlSi18Cu3CrMn + P (T6, water 20 °C)	0.6	4.9 × 10^−4^	2.0 × 10^3^	5.5	45.8 × 10^−4^	0.22 × 10^3^	
counterbody	5.0	40.1 × 10^−4^	0.25 × 10^3^				
AlSi18Cu3CrMn + P, Ti, B, Be (T6, water 20 °C)	0.3	2.5 × 10^−4^	4.0 × 10^3^	3.3	27.0 × 10^−4^	0.37 × 10^3^	
counterbody	3.0	24.5 × 10^−4^	0.4 × 10^3^				
AlSi18Cu3CrMn + P, Ti, B, Be (T6, water 50 °C)	0.4	3.3 × 10^−4^	3.0 × 10^3^	1.3	10.6 × 10^−4^	0.94 × 10^3^	
counterbody	0.9	7.4 × 10^−4^	1.4 × 10^3^				

**Table 6:** Corrosion rates of AlSi18Cu3CrMn alloy in 1 M NaCl.

Alloy	72 h CR, g/(m^2^·h)	240 h CR, g/(m^2^·h)	360 h CR, g/(m^2^·h)
AlSi18Cu3CrMn + P (T6, water 20 °C)	0	0	0.00015
AlSi18Cu3CrMn + P, Ti, B, Be (T6, water 20 °C)	0.0014	0.00015	0.00015
AlSi18Cu3CrMn + P, Ti, B, Be (T6, water 50 °C)	0	0.00015	0.00014

**Table 7:** Corrosion rate results of AlSi18Cu3CrMn alloy in 1 M H_2_SO_4_.

Alloy	72 h CR, g/(m^2^·h)	240 h CR, g/(m^2^·h)	360 h CR, g/(m^2^·h)
AlSi18Cu3CrMn + P (T6, water 20 °C)	0.0132	0.0126	0.0126
AlSi18Cu3CrMn + P, Ti, B, Be (T6, water 20 °C)	0.0128	0.0123	0.0124
AlSi18Cu3CrMn + P, Ti, B, Be (T6, water 50 °C)	0.0133	0.0126	0.0136

## Data Availability

The original contributions presented in this study are included in the article. Further inquiries can be directed at the corresponding authors.
